# Egg-in-Cube: Design and Fabrication of a Novel Artificial Eggshell with Functionalized Surface

**DOI:** 10.1371/journal.pone.0118624

**Published:** 2015-03-13

**Authors:** Wenjing Huang, Fumihito Arai, Tomohiro Kawahara

**Affiliations:** 1 Department of Biological Functions Engineering, Kyushu Institute of Technology, Wakamatsu-ku, Kitakyushu, Japan; 2 Department of Micro-Nano Systems Engineering, Nagoya University, Chikusa-ku, Nagoya, Japan; New York Medical College, UNITED STATES

## Abstract

An eggshell is a porous microstructure that regulates the passage of gases to allow respiration. The chick embryo and its circulatory system enclosed by the eggshell has become an important model for biomedical research such as the control of angiogenesis, cancer therapy, and drug delivery test, because the use of embryo is ethically acceptable and it is inexpensive and small. However, chick embryo and extra-embryonic blood vessels cannot be accessed freely and has poor observability because the eggshell is tough and cannot be seen through, which limits its application. In this study, a novel artificial eggshell with functionalized surface is proposed, which allows the total amount of oxygen to pass into the egg for the chick embryo culturing and has high observability and accessibility for embryo manipulation. First, a 40-mm enclosed cubic-shaped eggshell consisting of a membrane structure and a rigid frame structure is designed, and then the threshold of the membrane thickness suitable for the embryo survival is figured out according to the oxygen-permeability of the membrane structure. The designed artificial eggshell was actually fabricated by using polydimethylsiloxane (PDMS) and polycarbonate (PC) in the current study. Using the fabricated eggshell, chick embryo and extra-embryonic blood vessels can be observed from multiple directions. To test the effectiveness of the design, the cubic eggshells were used to culture chick embryos and survivability was confirmed when PDMS membranes with adequate oxygen permeability were used. Since the surface of the eggshell is transparent, chick embryo tissue development could be observed during the culture period. Additionally, the chick embryo tissues could be accessed and manipulated from outside the cubic eggshell, by using mechanical tools without breakage of the eggshell. The proposed “Egg-in-Cube” with functionalized surface has great potential to serve as a promising platform for biomedical research.

## Introduction

Recently, tightening of regulations for animal experiments due to ethical problems has limited their use in research; however, animal models are still essential because progress in the biomedical research is heavily dependent on suitable in vivo models. Therefore, scientists often employ embryos such as those of chicks, zebrafish, and Xenopus laevis embryos as alternatives to the use of animals in biomedical research [1–5]. The applicability of an embryo model for animal experiments and human risk assessment has increased dramatically since the annotation of the genomes of several modern organisms; for example, the genome of zebrafish shares a high degree of sequence similarity to that of humans. Therefore, the results obtained from an embryo model confer a high predictability of the occurrence of diseases in vertebrates, including humans [6–9].

Among the commonly used embryo models, the chick embryo is a preferred alternative to animal models for experiments. For example, the chick embryo has been used extensively as a model system for studying angiogenesis since its development is accompanied by the formation of new blood vessels on the chick chorioallantoic membrane (CAM), an extra-embryonic respiratory organ of the chick embryo [10, 11]. Experiments using chicken eggs usually focus on the CAM or the embryo tissue, and this model has several advantages compared to other animal models. First, ethically acceptable since the chick embryo is an experiment model containing no functional nerves before embryonic day 10, therefore, tissues, including human tissue and cells, can be transplanted on the surface of the CAM without causing transplant rejection or inflicting any pain on the embryo [12, 13]. Second, chick embryos are less costly than other experimental animals. Fertilized eggs can be purchased for less than 1 USD/egg from a local market. In addition, because the egg yolk acts as a source of nutrients, no culture medium is required for the growth of a chick embryo until hatching. Third, these embryos show a high degree of usability, as the chick embryo has a short life cycle that provides rapid experimental results [14]. In addition, since the chick embryo does not escape from the egg, the experimental procedure could be completed easily without anaesthesia that is required when using other animal models [15].

Chick embryos possess two extra-embryonic blood circulatory networks that act as a respiratory system: vitelline circulation that occurs over the surface of the yolk and plays an important role in the absorption of food, water, and oxygen while the embryo is in its earliest stage of development; and chorioallantoic circulation on the CAM when the chick embryos need more oxygen than can be supplied by only the vitelline circulation [16]. The CAM is pressed against the inside of the eggshell to absorb oxygen passing through the pores of the eggshell when the chick embryo becomes larger [11, 17–19]. Therefore, since oxygen supply is crucial for the development of chick embryos [20], it is necessary to appropriately control the oxygen permeation required for the respiratory system of chick embryos when applying the CAM as an animal model.

Although chick embryo culture has been used extensively in experiments as a promising system for biomedical research, one major problem remains in that the embryo enclosed by the eggshell is not directly observable. Traditionally, scientists have cut a small window in the eggshell over the air cell to observe the development of the chick embryo, but this method heavily limits the observation and accessibility of the tissue and vascular networks [21–23]. To overcome this limitation, several attempts have been made to develop artificial eggshells. It is important to note that a chick embryo should be transplanted to the artificial eggshell within three days from the start of the incubation (before formation of the blood capillary network) to prevent damage induced by cracking of the eggshell and improve the embryo survival rates [20, 24, 25]. New et al. and subsequent groups (e.g., Gallera & Nicolet et al.), isolated the blastoderm of the chick embryo from the vitelline membrane on the yolk in the early stage when the embryo was very fragile and cultured it using a glass dish [26–28]. Kamihira et al. developed an artificial culture vessel for quail embryo cultures using a Teflon membrane, the shape of which was supported by a stainless steel mesh in a glass cup [20]. Although these methods allowed effective observation of embryo development through the highly transparent shell, it was nonetheless difficult to access the chick embryos freely because the shell was composed of rigid glass (high stiffness) and fragile film (low stiffness). As a functional platform for tissue and blood vessel manipulation, artificial eggshells should have specifically-designed surface possessing not only good transparency but also appropriate rigidity.

We notice that the selection of an appropriate animal model for biomedical research should consider not only factors such as cost, availability and ethical implications but also the ease and adaptability for experimental manipulation [29]. Moreover, for the manipulation of animal models, the angles and locations of the manipulating instruments are important factors [30, 31]. Therefore, a new type of eggshell for easy embryo manipulation is required.

Hence, we aimed to develop a novel and easy-to-handle artificial eggshell with functionalized surface to enable observation and manipulation of a chick embryo from multiple directions, which is a requirement for biomedical research. Since functionalized membrane surface is deformable, to achieve our goal, we integrated a rigid frame structure to fabricate the eggshell, with self-sustaining and shape-retaining functions. An embryo containing yolk and albumen was transferred into the artificial eggshell, and chick embryos grew well in the enclosed artificial eggshell by controlling the oxygen permeation rate based on the design method. Because of the durability of the eggshell, tissues and blood vessels of the embryo can be observed at almost any point and could be accessed freely without making a cleft gate on the membrane surface.

## Materials and Methods

### Ethical approval

According to the Scientists Center for Animal Welfare (SCAW) guidelines, studies on embryonated eggs are included in Category A of biomedical experiments, which has the lowest ethical concerns [32]. According to the Animals (Scientific Procedures) Act 1986, a chick embryo in its embryonic form is considered a protected animal only from the stage of development at which half of the hatching period has elapsed; therefore, experiments on chick embryos before embryonic day 10 pose few ethical problems [33]. Considering these ethical concerns, in the current study, the incubation period was set to less than 10 days, and experiments were performed only on chick embryos aged less than 10 embryonic days. Based on this regulation, the present study was approved by the Ethics Committee of the Graduate School of Life Science and Systems Engineering (LSSE) at Kyushu Institute of Technology.

### Required specifications

The two primary desired properties for the proposed artificial eggshell with functionalized surface are as follows:

1. Culturing: a chicken eggshell has a porous structure permitting the free flow of oxygen through the shell [34]. Oxygen is particularly important for the development of the circulatory system and the chick embryo because cells have limited oxygen stores compared to metabolic substrates such as glucose and amino acids [35]. Therefore, similar to a natural chicken eggshell, an　artificial eggshell should possess high oxygen permeability and biocompatibility for appropriate culturing.
2. Observation and operation: as discussed above, transparency and appropriate rigidity of the artificial eggshell are necessary requirements to achieve both high observability and accessibility functions.

### Design of the artificial (cubic) eggshell

**Fig 1:**
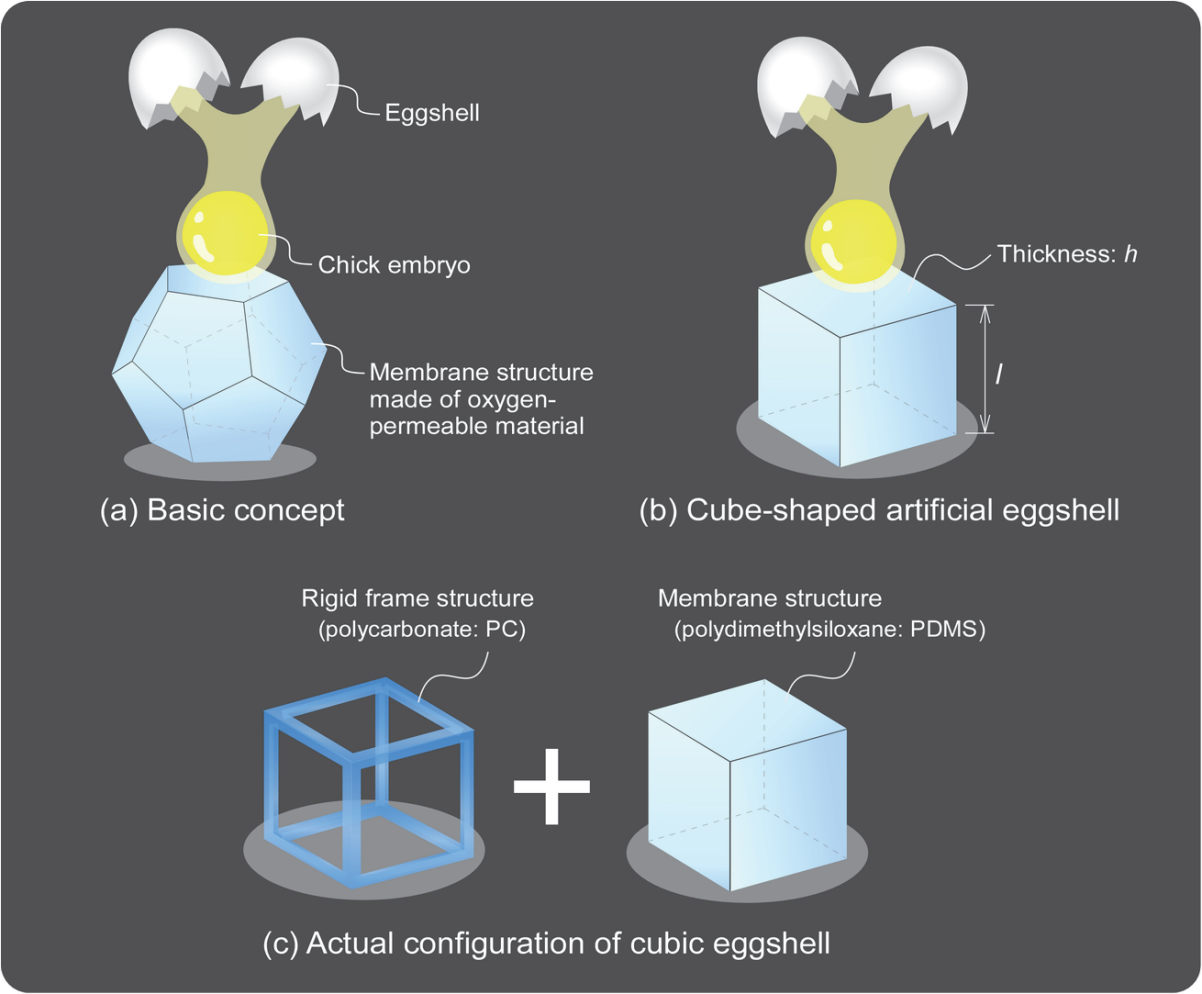
Conceptual images of cubic eggshell using a chick embryo. (a) The artificial eggshell is transparent, polyhedral-shaped, and fabricated from an oxygen-permeable material. Contents of the chick egg are transferred into the eggshell, and the developed embryo can be observed and operated from multiple directions. (b) A cube-shaped eggshell fabricated using oxygen-permeable membranes of thickness *h* with a side length *l*; its fabrication is easier than that of polyhedral-shaped eggshells and its posture (observation point) can be changed easily. (c) A rigid frame structure is introduced into the cubic eggshell to maintain the shape of the eggshell.

Fig. 1 shows the basic concept and artificial eggshell design based on the required specifications for culturing, observation and operation of chick embryos described above. We initially intended to design a polyhedral-shaped eggshell to most closely mimic the shape of the normal eggshell (Fig. 1 (A)) using a thin membrane surface with oxygen permeability and transparency. However, it is difficult and costly to fabricate such a high-dimensional polyhedron structure. Therefore, we next considered a cube-shaped eggshell (Fig. 1(B)), which is easier to fabricate and whose posture (observation point) can be changed easily. Further, we noticed that artificial eggshell fabricated with only thin membranes is not a self-supporting structure due to the lack of structural rigidity. Therefore, as shown in Fig. 1(C), we integrated a rigid frame in the cubic eggshell, which helped to maintain its shape.

As mentioned above, oxygen permeation is an important factor for the survival of a chick embryo in an artificial eggshell. The permeation coefficient of oxygen in a membrane is expressed as shown in Equation (1) [36]:

$$\alpha =\frac{V_{c}h}{\tau S\Delta p}$$ (1)

where *V_c_* is the volume of oxygen permeating in a membrane with a thickness of *h* during a time period of τ, *S* is the effective oxygen-permeable area of the membranes, and Δ*p* is the pressure difference converted from the oxygen concentration difference between the two sides of a membrane.

In order to realize the above-mentioned design, we used polydimethylsiloxane (PDMS) as a material of membrane structure. PDMS is optically clear and has been extensively used in cell culture because of its high biocompatibility [37]. Since the mechanical strength (Young’s modulus) of the PDMS membrane is thickness-dependent [37], the desired rigidity for embryo manipulation can be obtained by appropriate design. Most importantly, PDMS is a material that possesses thickness-dependent oxygen permeability [38–41]. Therefore, it is necessary to apply a PDMS membrane of appropriate thickness to achieve good oxygen permeability for development of the chick embryo. In order to support the shape of a cubic eggshell, a cubic frame made of polycarbonate (PC), which has a Young’s modulus of approximately 2 GPa was selected in the current study. According to previous studies, the permeation coefficient of oxygen, *α*, in PDMS membrane is equal to 3 ×10^–11^ (cm^3^∙cm)(cm^−2^∙sec^−1^∙Pa^−1^) [39, 40].

Based on the above information, we designed the cubic eggshell by considering the oxygen volume necessary for survival of a chick embryo. A chicken egg takes 21 days to develop and hatch, and an embryo consumes approximately 6 L of oxygen during this period [42]. Therefore, we supposed that *V_c_* = 286 mL of oxygen is necessary for a chick embryo per day. According to this calculation, Δ*p* = 101.33 kPa and τ = 86400 sec. Assuming an egg volume of 50 mL [43], we designed a cubic eggshell with a side length of *l* = 40 mm. From the side length, we calculated the total area of the PDMS membrane, and determined that *S* should be 9600 mm^2^. Therefore, to achieve sufficient oxygen permeation for the normal development of a chick embryo, according to Equation (1), the thickness of the PDMS membrane, *h*, should be

$$h=\alpha \frac{\tau S\Delta p}{V_{c}}<0.88\;mm.$$

According to this result, in order to ensure that the volume of permeating oxygen is sufficient for proper embryo development, a very thin PDMS membrane should be used with a Young’s modulus that is quite small (several hundred kilopascals). However, under these conditions, the cubic eggshell cannot support the weight of the contents of an egg independently and maintain its shape. To solve this problem, an inner frame having sufficient strength and high rigidity is required. In this study, the thickness of the beam of the PC frame structure was designed to be 4 mm to achieve sufficient strength. The application of the frame structure leads to a 40% reduction in the effective oxygen-permeable area. Therefore, the final thickness of the PDMS membrane for the cubic structure should be

h<0.55mm. (2)

**Fig 2:**
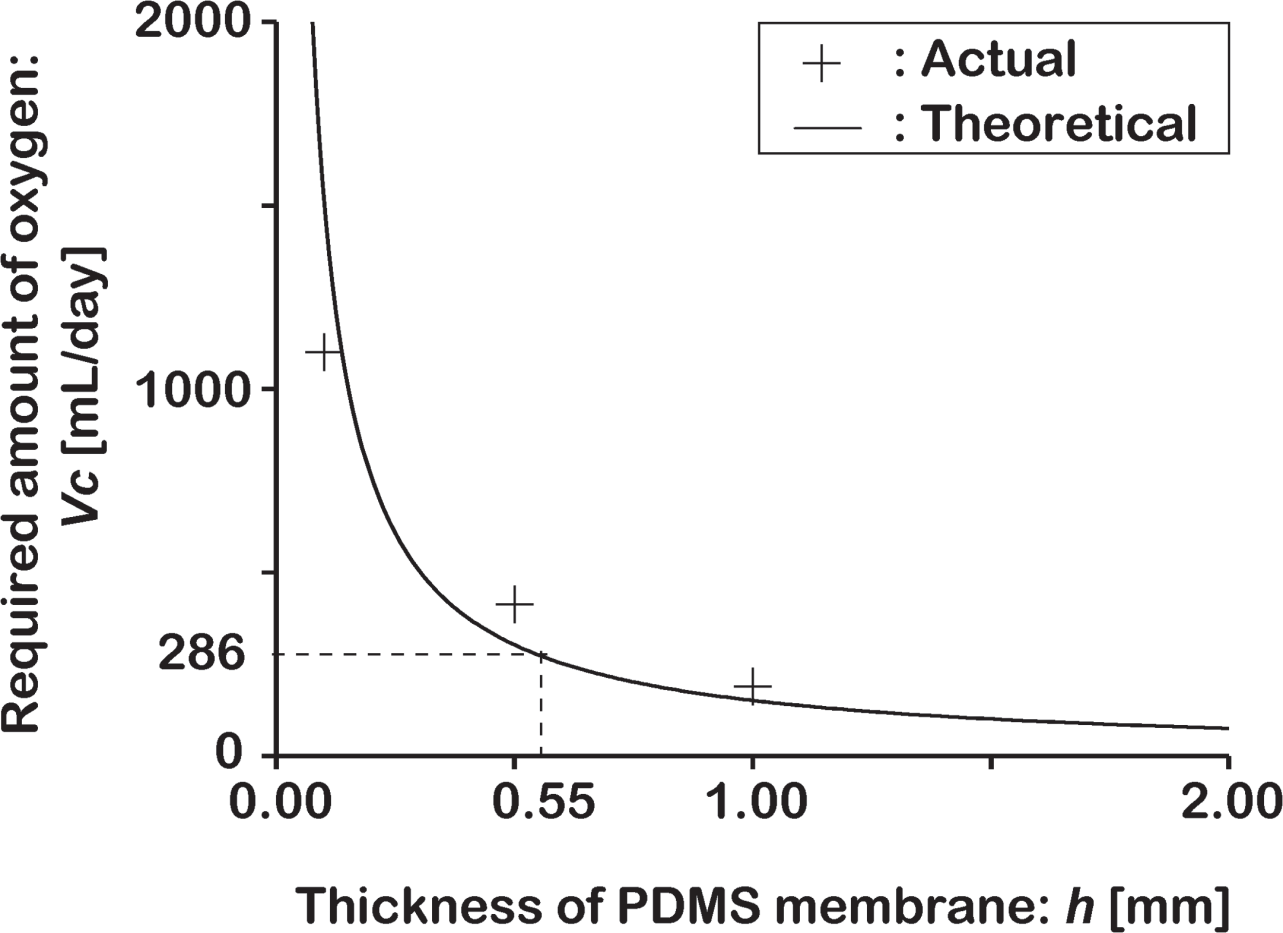
Required oxygen permeation rate in the PDMS membrane for chick embryo culturing. The permeating rates of 0.1-, 0.5-, and 1.00-mm-thick PDMS membranes were measured with an oxygen permeation analyzer, and the normalized results were 1150, 413, and 191 mL/day, respectively. In the current study, the lowest oxygen volume for embryo survival was estimated to be 286 mL/day (corresponding to a membrane thickness 0.55 mm), according to the total oxygen volume (6 L) consumed by a chick embryo during the embryonic period (21 days) [42].

Fig. 2 shows an estimated curve of oxygen permeation in PDMS membranes of different thickness, which was calculated from Equation (1). The measured data for oxygen permeation were obtained from an oxygen permeation analyzer (Oxygen Permeation Analyzer 8001, Systech Illinois Inc.), which matched the estimated values well. From this figure, we can estimate the effectiveness of the thickness of the PDMS membrane for culturing. The effectiveness of the thickness of the PDMS membrane surface was then confirmed experimentally.

### Fabrication and preparation of the cubic eggshell

The fabrication process of the cubic eggshell and the PDMS membranes with different thickness used in the current study are shown in S1 Fig. and S2 Fig. in Supporting Information, respectively.

Briefly, the cubic eggshell was fabricated by integrating a PC frame structure and six PDMS membranes, with an unsealed upper face. The contents of 3-day-cultured chick embryos were then transferred to the eggshell following previously described methods [31]. In the current study, fertilized chick eggs (Sakura, Gotofuranjyo Inc.) were used. Subsequently, the upper face of the cubic eggshell was sealed using liquid PDMS as glue. Finally, the chick embryos were incubated at 39°C with a relative humidity of 80%.

**Fig 3:**
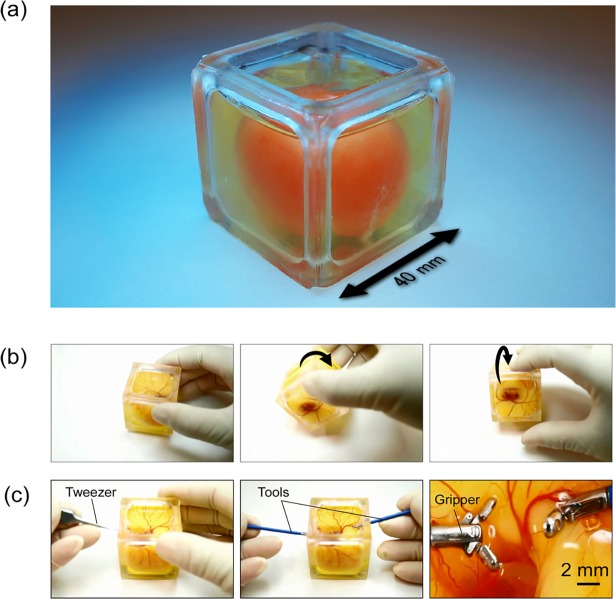
Developed cubic eggshell and the observation and manipulation of chick embryo. (a) Overview of the fabricated cubic eggshell with a chick embryo. The membrane thickness is 0.3 mm, the length of one side is 40 mm, and the total volume of the cubic eggshell is approximately 50 mL. (b) Posture change of the chick embryo in the cubic eggshell. It is possible to observe the chick embryo from multiple directions by changing the posture of the chick embryo. (c) Manipulation of the chick embryo in the cubic eggshell fabricated using 0.3-mm-thick PDMS membranes. Holes were made with tweezers, and then small grippers with a diameter of 2 mm were inserted inside the shell to manipulate the tissues of the chick embryo under the microscope.

An overview of the fabricated cubic eggshell with a chick embryo is shown in Fig. 3. The use of accessible material and ease of the fabrication process could contribute to mass production of eggshells and reduction of fabrication costs (low-cost and disposable).

In order to confirm the usability of the developed shell, demonstrations were conducted as shown in S1 and S2 Movies. In this experiment, the chick embryos were cultured for 7 days in a shell with a membrane thickness of 0.3 mm. The results of Fig. 3(B) confirmed that if a cube-shaped shell is used, it is easy to change the posture (observation angle) of the chick embryo to find the desired observation point (S1 Movie). Fig. 3(C) shows the mechanical manipulation of the chick embryo. In this experiment, holes were made in the shell with a pair of tweezers. Then, small grippers (tools) with a 2-mm diameter were inserted in the shell. Finally, the tissue and blood vessels of the chick embryo were manipulated using the tools under the microscope. Through this experiment, we confirmed that the functionalized PDMS membrane was maintained during the manipulation owing to its good mechanical strength. Therefore, the tool insertion points were not cracked and there was no leakage observed during the manipulation process (S2 Movie).

### Embryo culture

During the experiment, we stopped the embryo culture according to the developmental stage and number of the embryonic days recommended with respect to ethical concerns. It has been reported that, on embryonic day 7 (Hamburger-Hamilton stages [HH] 31), chick embryos possess the following developmental properties: feather germs on the dorsal surface and thigh and a body size greater than 2 cm [14, 44, 45]. Here, we cultured the chick embryos for up to 7 days (HH31), at which point feather germ formation was minimal. Therefore, egg turning was not conducted during the culturing, although the cube-shaped eggshell is suitable for turning, because the experiments were not conducted until hatching.

### Image acquisition and judgment of survivability

For chick embryos cultured in cubic eggshell, photos and movies were taken every 24 hours using the video mode of a digital camera (NEX-5TL, Sony Inc.).

The chick embryo was regarded as alive based on the following properties; 1) it moved around when imaged, 2) beating of the heart could be observed when imaged, and 3) the heart and blood vessels were red. The normal development of the extra-embryonic circulatory system such as blood vessel networks (branches) and large vessels could be confirmed. On the other hand, the chick embryo was regarded as dead when the following phenomenon were observed; 1) no movement of the chick embryo or heart beat was detected, 2) the extra-embryonic circulatory system was broken, and 3) the contents of cubic eggshell, including chick embryo, albumen, and yolk, appeared to be rotting and had a soup-like texture.

To further investigate the survivability of chick embryos over a range of developmental stages in cubic eggshells, movies of both chick embryos in normal eggshells with a hole in the air chamber and cubic eggshells made of 0.1-, 0.3-, and 0.7-mm-thick PDMS membranes were obtained using a digital camera (GX-FW-28S5C-C, ViewPLUS Inc.) mounted on a stereo microscope (Leica S8 APO, Leica Microsystems Inc.), and the heart rate of the chick embryo was analyzed using the grayscale images from the movies. From these images, we found that the gray value in the cardiac chamber changed cyclically because of the cyclical movement of blood flow. Therefore, in order to calculate the heart rate, the average gray value of a rectangular area including the cardiac chamber was calculated using Matlab R2013a with Image Processing Toolbox, a method used in the previous study [46] (S3 Fig.), and results of the analysis were confirmed by manually tracing the movement of the blood flow.

### Statistical analysis

All data were expressed as mean ± SEM. Differences of the crown-to-rump length and one eye size between chick embryos in normal eggshells and cubic eggshells were compared each day during the culture period using Student t-test. A value of *P* <0.05 was considered to be statistically significant.

## Results

### Survivability of chick embryos

**Fig 4:**
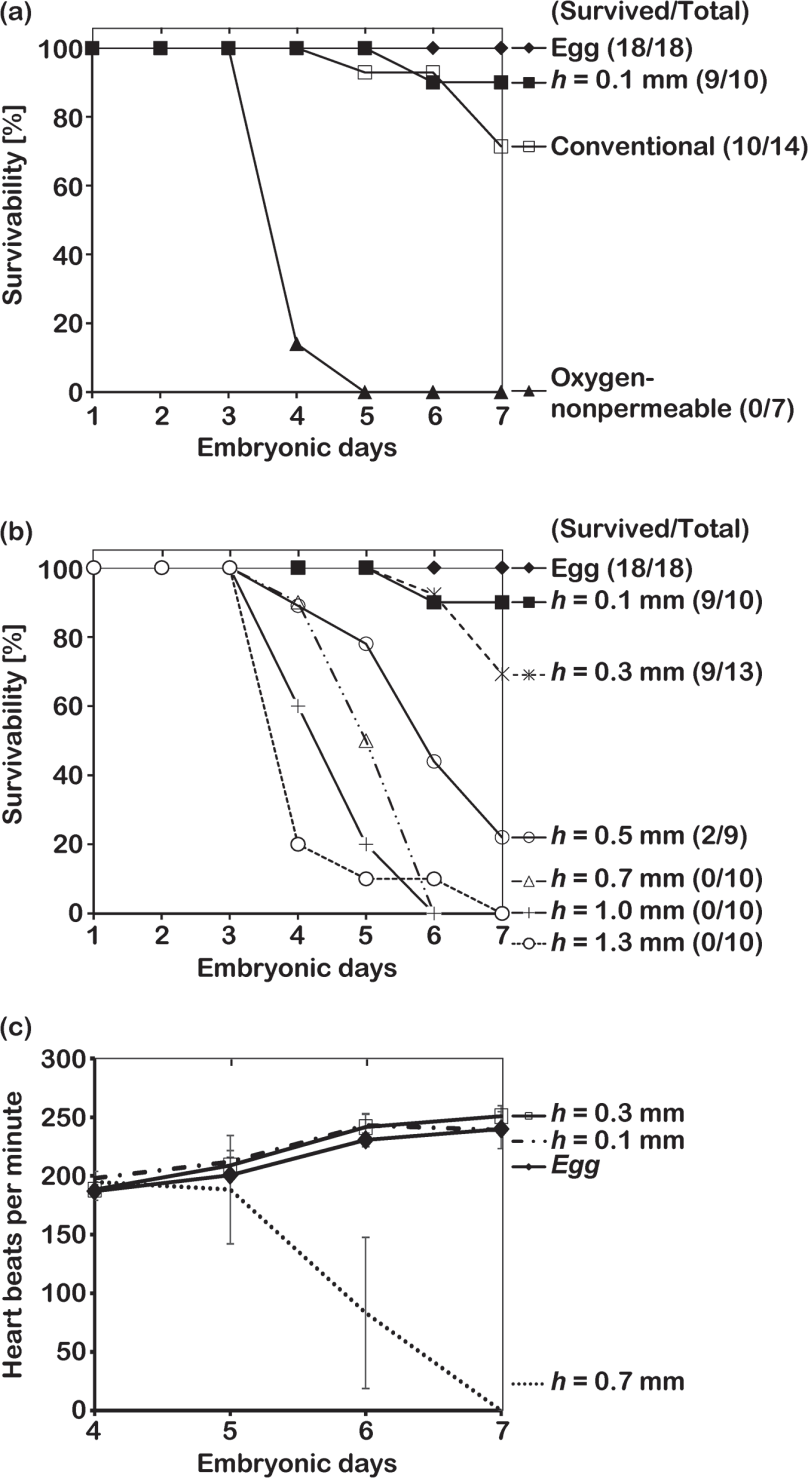
Survival rates of chick embryos cultured under different conditions. (a) Effect of the environment change. The survivability of chick embryos in the normal eggshell was 100%. The survivability of chick embryos in cubic eggshells fabricated using 0.1-mm-thick PDMS membranes (90%) was higher than that of the conventional culture method (71%). The chick embryos cultured in cubic eggshells without oxygen permeation died soon after transfer. (b) Effect of the thickness change. On day 7, the survivability of chick embryos in cubic eggshells fabricated using 0.3- and 0.5-mm-thick PDMS membranes was approximately 80% and 22%, respectively. Chick embryos in eggshells using 0.7-, 1.0-, and 1.3-mm-thick membranes died soon after transfer. This tendency is consistent with the oxygen permeation rate calculated using Equation (1). (c) Heart rates of the chick embryos in normal eggshells and cubic eggshells fabricated using 0.1-, 0.3-, and 0.7-mm-thick membranes. Heart rates of chick embryos in cubic eggshells fabricated using 0.1-mm-thick membranes increased from 198 ± 6 to 239 ± 16 beats per minute, and eggshells using 0.3-mm-thick membranes increased from 188 ± 5 to 250 ± 9 beats per minute. The results were similar to the heart beats of chick embryos in normal eggshells. On the other hand, the average heart rates of chick embryos in eggshells using 0.7-mm-thick membranes decreased dramatically from day 5 because of the death of some chick embryos, which resulted in a high variance. The error bar is SEM (n = 5).

In order to evaluate the validity of our design, chick embryos were cultured in the developed cubic eggshell. We investigated the survivability of thick embryos by calculating the number of days that the survivability of embryos could be confirmed (Fig. 4). For comparison, we also cultured chick embryos in a plastic cup covered by a 10-μm-thick film (conventional method as a control model) and in the cubic eggshell covered by a 1-mm-thick polystyrene plate (non-permeable model) (Fig. 4(A)). We found that the survival rates of normal eggshells were approximately 100% during the culture. According to the results, we identified that the incubation conditions employed, such as the temperature and humidity were appropriate for chick embryo development. On the other hand, the survival rate was approximately 90% for the incubation using the cubic eggshells with 0.1-mm-thick membranes, which was higher than that observed for the 10-μm-thick film (71%). However, the chick embryos died soon after we transferred the contents of the eggs into the cubic eggshells in the no oxygen permeability model. These results strongly suggested that oxygen permeation through the cubic eggshells is important for the survivability of chick embryos, and indicated that the PDMS membrane could be an excellent candidate for the cubic eggshell, similar to the thin plastic films used in previous studies.

Since oxygen permeability is dependent on the thickness of the PDMS membrane, we also tested the effects of membrane thickness on the survivability of chick embryos. As shown in Fig. 4(B), the survival rate of chick embryos using 0.3-mm-thick membranes was approximately 80%, which was lower than that obtained with 0.1-mm-thick membranes. In addition, all of the chick embryos in cubic eggshells fabricated with 0.7-, 1.0-, and 1.3-mm-thick membranes died before day 7. According to Equation (1) shown under the Design of the artificial (cubic) eggshell in this article, 0.5-mm-thick membranes should provide the threshold oxygen content for chick embryo development; accordingly, we found that approximately 22% of chick embryos in the 0.5-mm-cubic eggshell stayed alive until day 7. In addition, as shown in Fig. 4(C), the heart rates of chick embryos in cubic eggshells fabricated using 0.1- and 0.3-mm-thick PDMS membranes increased with the development of the chick embryos from day 4 to day 7, which were similar to the increase tendency of the heart rates of the chick embryos in normal eggshells. On the other hand, the heart rates of chick embryos in cubic eggshells fabricated using 0.7 mm decreased dramatically on day 5 and became zero before day 7. These results demonstrated that appropriate design of PDMS membrane thickness is crucial for oxygen permeation and therefore important for the development of chick embryos. On the other hand, since the mechanical strength of the PDMS membrane is also dependent on its thickness, the membrane strength should also be considered when developing the eggshell. In this study, we found that chick embryos grew well in cubic eggshells fabricated with 0.1- and 0.3-mm-thick PDMS membranes, as estimated from Equation (2) shown under the Design of the artificial (cubic) eggshell in this article. However, when we inserted instruments into the cubic eggshells, we found that eggshells fabricated with 0.3-mm-thick, but not 0.1-mm-thick, membranes stayed in good condition and had no albumen leakage, as shown in Fig. 3(C) and S3 Movie. Overall, these results suggest that our approach used to design and fabricate the cubic eggshell with a 0.3-mm-thick membrane surface is appropriate to realize high accessibility with 7 days of culturing.

### Development of chick embryos

**Fig 5:**
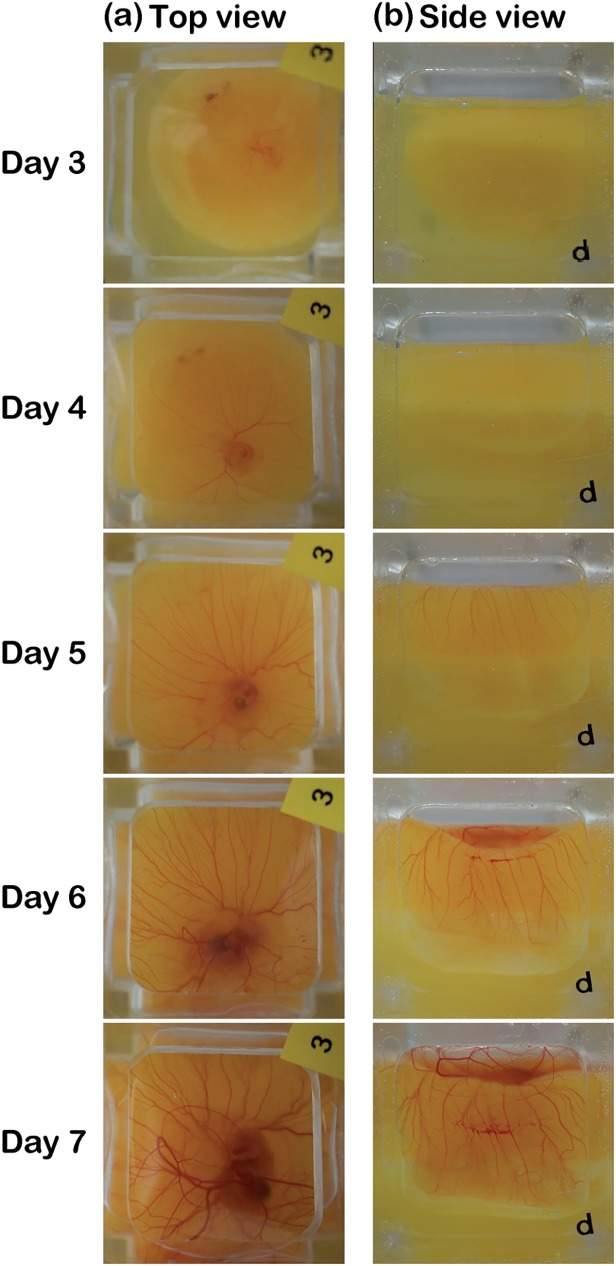
Formation of blood vessels on the top and a side membrane of a cubic eggshell. (a) Top views show that the blood vessel network grew gradually, and the embryo became increasingly larger with the development of its circulatory system. (b) Side views show that when the body of a chick embryo became large, the extra-circulatory system spread onto the side membrane to search for a larger volume of oxygen.

Blood vessel formation on the faces of the cubic eggshell fabricated using 0.3-mm-thick PDMS membranes could be observed simply without requiring a specific apparatus, as shown in Fig. 5. As shown in Fig. 5(A) and (B), from the top face, we found that the chick embryo increased in size gradually following egg cracking, and similarly, the blood vessel network became increasingly complicated and spread rapidly from day 3 to 7. There were no blood vessels observed on the lateral membrane after the egg contents were transferred into the cubic eggshell on day 3. By contrast, after 7-day development, the blood vessels spread almost all around the lateral-side membrane. These results suggest dramatic development of the circulation system of the chick embryo in the cubic eggshell, which indicates that the phenomenon of extensive blood vessel formation on the inner surface of normal eggshells to absorb oxygen passing through the pores of the eggshell could be observed in the cubic eggshell. Furthermore, when we observed the sidewall of the eggshell, not only the distribution of blood vessels, but also the flow of the blood cells was confirmed, as shown in S4 Movie.

**Fig 6:**
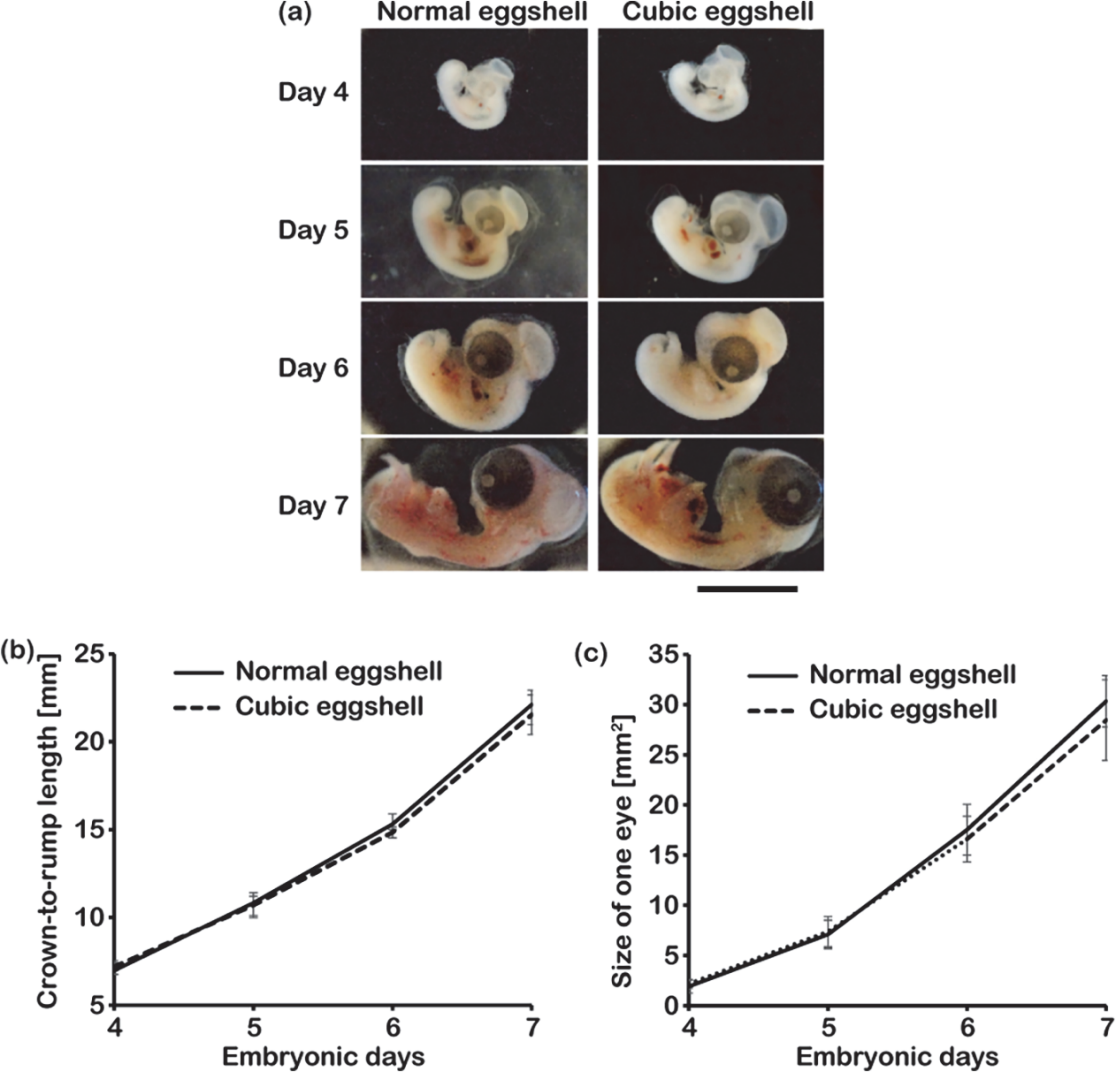
Development of chick embryos in cubic eggshell and normal eggshell. (a) Left: chick embryos developed in normal eggshells from day 4 to 7. The chick embryos developed to HH31, according to the properties of their body such as egg tooth, body length, etc. Right: chick embryos developed in cubic eggshells fabricated using 0.3-mm-thick PDMS membranes. The body properties, including egg tooth and body length, were comparable to those of embryos in the normal eggshell. Scale bar for (a): 10 mm. (b) The crown-to-rump length of chick embryos in normal eggshells and cubic eggshells fabricated using 0.3-mm-thick PDMS membranes. From day 4 to 7, there was no significant difference. (c) The eye size of the embryos from day 4 to 7. There was also no significant difference between the embryos cultured in normal eggshells and cubic eggshells. The error bar is SEM (n = 5).

In order to compare the development of chick embryos cultured in normal eggshells and the cubic eggshells, we examined their anatomical features over a range of developmental stages. From day 4 to day 7, the chick embryo was transferred to a fresh Petri dish filled with cold, sterile phosphate buffer saline (PBS) and the surrounding membranes were carefully torn apart with fine forceps. Typical images of chick embryos in the normal and cubic eggshell are shown in Fig. 6(A). On day 7, embryos in normal and cubic eggshells had very conspicuous eyes and the egg tooth could be observed on the tip of the beak. All organs were formed and the heart was completely inside the chest. These features demonstrate that the chick embryos successfully developed to HH stage 31. To quantitatively analyze the embryo development, the crown-to-rump length and eye size of the chick embryos in normal eggshells and cubic eggshells fabricated using 0.3-mm-thick PDMS membranes were measured from the images of embryos using ImageJ 1.48v (NIH). As shown in Fig. 6(B), the crown-to-rump length of chick embryos in normal eggshells grew from 7.0 ± 1.1 mm to 22.1 ± 1.7 mm in normal eggshells, and from 7.2 ± 0.5 mm to 21.5 ± 2.3 mm in cubic eggshells. No significant difference was observed. Also, the eye size of chick embryos in normal eggshells and cubic eggshells had a similar increase tendency on day 4, 5, 6, and 7 (Fig. 6(C)). Although the eye size of the chick embryo from the cubic eggshell was slightly smaller than that in the normal eggshell (Fig. 6(C)), the difference was not significant. This means that, at least until embryonic day 7, the developed cubic eggshell made of PDMS membranes with appropriate membrane thickness shows suitable performance for chick embryo culture.

### Inducing blood vessel formation

**Fig 7:**
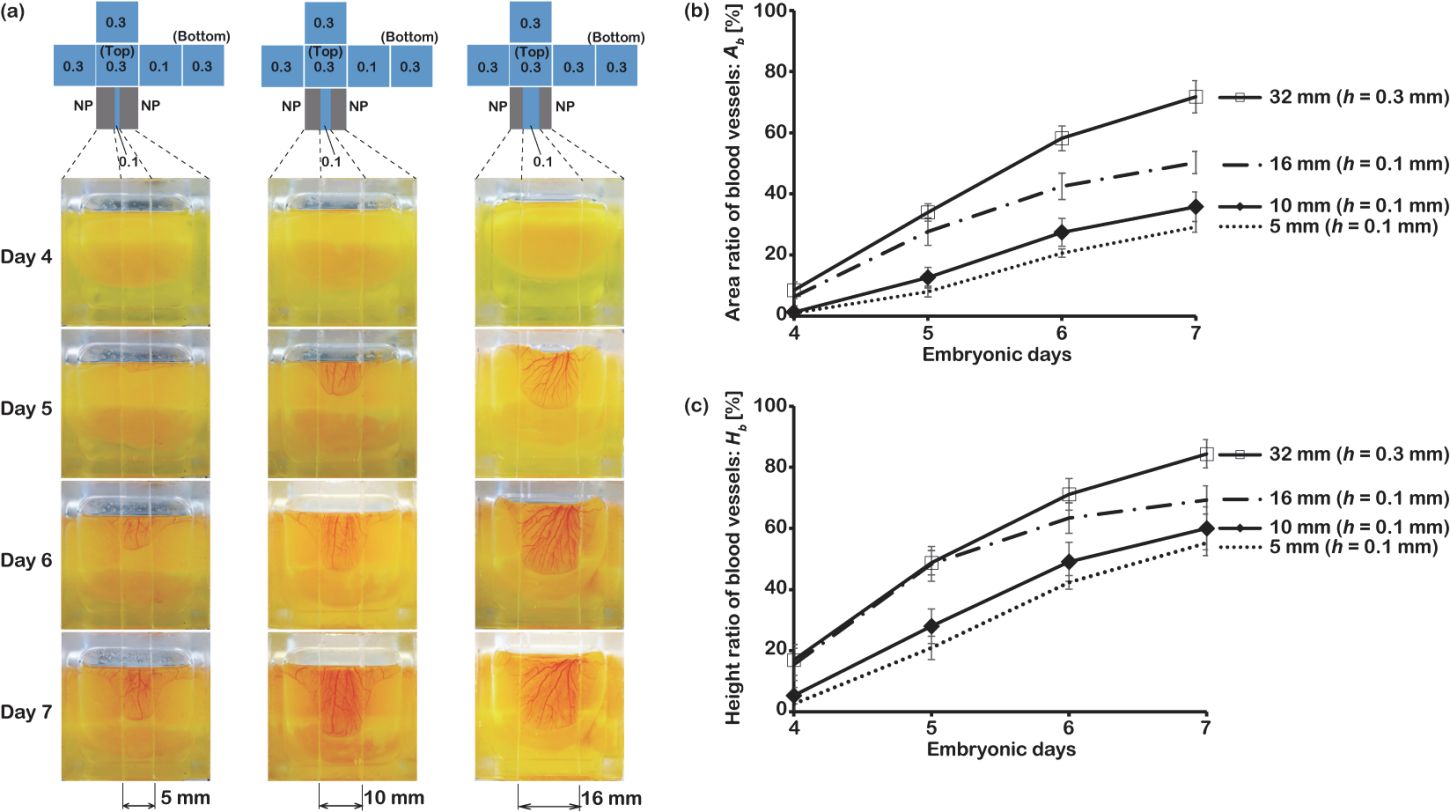
Spatial control of embryo blood vessel formation on the patterned side membranes. The total oxygen volume permeating into each cubic eggshell was calculated to be comparable to that of cubic eggshells fabricated from six 0.3-mm-thick PDMS membranes. (a) Typical images of blood vessel formation in oxygenated channels with widths of 5, 10, and 16 mm, respectively. Polystyrene plates were glued on a 0.1-mm-thick PDMS membrane to make an oxygen non-permeable (NP) area. From day 4 to 7, blood vessels grew selectively in the oxygen-permeable channels. (b) Area ratios of blood vessels on a whole lateral-side membrane (width: 32 mm) made from a 0.3-mm-thick membrane and in the channels with widths of 5, 10, and 16 mm (0.1-mm-thick membrane). From day 4 to 7, the area of blood vessel formation decreased prominently in the narrow channel because of the decrease of oxygenated area on the patterned lateral-side membrane. (c) Height ratios of the area of blood vessel formation from day 4 to 7. On a whole lateral-side membrane or in the oxygenated channel with a width of 16 mm, blood vessels spread rapidly after the transferring of egg contents. On the other hand, blood vessels grew slowly in the oxygenated channel with a width of 5 mm. The error bar is SEM (n = 5). The detailed process of image analysis and definition of *A*_*b*_ and *H*_*b*_ are shown in S4 Fig.

An advantage of the developed cubic eggshell is that the thickness of the PDMS membrane (oxygen permeability) can be freely changed under the conditions of Equation (1) shown under the Design of the artificial (cubic) eggshell in this article. For example, the permeating oxygen volume can be adjusted to an appropriate level for chick embryo development by changing the thickness of the membranes and oxygen non-permeable plates. In this experiment, in order to figure out the relationship between the oxygen permeable area and the growth of blood vessels on the lateral-side membranes, the lateral-side membranes of the cubic eggshell were fabricated using patterned membranes with oxygen permeable and non-permeable areas of different widths. We attached two polystyrene plates on the left and right sides of the lateral-side membrane to form two oxygen non-permeable areas as shown in Fig. 7(A). The middle area of the side face was a 0.1-mm-thick PDMS membrane with widths of 5, 10, or 16 mm. According to Equation (1) shown under the Design of the artificial (cubic) eggshell in this article, since there was a large decrease in the oxygen-permeating area on the face with a 5- or 10-mm-wide channel, we changed the membrane thickness of another side face from 0.3 mm to 0.1 mm, which ensured that the total oxygen permeating volume was comparable to that permeating in the eggshell fabricated using the 0.3-mm-thick PDMS membrane. In the channel with a width of 16 mm, since the oxygen permeating rate for 0.1 mm is more than twice as large as that for 0.3 mm, oxygen permeation will be relatively larger than that permeating over the whole face made of a 0.3-mm-thick membrane. Therefore, the total permeating oxygen volume of the patterned eggshell is comparable to that of the cubic eggshell fabricated using 0.3-mm-thick PDMS membranes. In order to clarify the changes of blood vessel formation in the channel with widths of 5, 10, and 16 mm, the blood vessel formation on the 0.3-mm-thick lateral-side membrane (width: 32 mm) was used as a control. From Fig. 7(A), we found that blood vessels formed selectively in the oxygen permeable channels made of the 0.1-mm-thick PDMS membranes. Since the lateral-side membrane was transparent and the bottom edge of the spreading blood vessel network could be clearly observed, we calculated the degree of vasculature using the area revealed by the bottom edge. In this study, we selected eggs with almost the same volume, and the spreading area and height of blood vessel network was divided by the area occupied by egg albumen and its depth, respectively. This quotient was defined as the area/height ratio. The detailed process of image analysis is shown in S4 Fig. Fig. 7(B) shows that the area ratio of blood vessel network decreased when the width of the oxygen permeable channel became narrow. In addition, as shown in Fig. 7(C), the growth speed of blood vessel network in the narrow channel was also slower than that in a wider one. These results suggest that, by precisely controlling the oxygen permeation on one surface with a designed pattern, we can create new functions such as regulation of the growth and spacing of new blood vessels. This result demonstrates promising applications of these eggshells for biomedical research, such as cardiovascular simulation.

## Discussion

In this study, we evaluated the survival rates of chick embryos over a 7-day incubation period. The average size of blood vessels on the chicken CAM on day 7 was similar to that of micro-vessels in human organs, such as the human brachial artery (<1.2 mm) and radial artery (<1 mm) [47]; therefore, the CAM could be a useful microvascular training model for surgeons and nurses. For instance, microsurgery for regions such as the brain, eyes, and joints requires small surgical tools, and surgical operations must be conducted under a microscope. Tiny blood vessels can be connected and treated by fine manipulation under the magnified field of operation. Since a high level of technical skill is required for surgeons to handle tiny tissues and blood vessels, surgical training by using an animal model is the most effective method to improve surgical performance [48]. In addition, the evaluation of microsurgical devices and robots with fine accuracy can be conducted using our platform to confirm the handling and suturing performance of tissue and blood vessels [49].

Recently, the chick embryo has become an important model in the fields of drug discovery, regenerative medicine, and cancer research, among others [50, 51]. For example, the cardiovascular system of the CAM is an attractive model for the evaluation of drug activity and pharmacokinetic profiles, since the effects of photosensitizing drugs (e.g., the regression of tumors) or the CAM intravascular fluorescence can be monitored in real-time mode by microscopy [50, 52, 53]. However, fluorescent material injection is limited for previous chick embryo models contained in chicken eggshells or recipient eggshells, which are not suitable for multi-directional real-time observations at high resolution. The cubic artificial eggshell with functionalized surface demonstrated in this study overcomes these problems, offers the possibility to observe and measure the blood flow of vascular network composed of very small vessels, and can contribute to building an effective experimental model with both in vivo and in vitro features for biomedical research such as drug delivery testing.

In the experiments, we succeeded in culturing chick embryos with an approximate 80% success rate until day 7 using both the conventional approach and the newly proposed approach (0.3-mm-thick membrane), and when we tried to grow embryos beyond this age (10 days, before they changed to a more painful and distressful category [32]) using cubic eggshell fabricated using 0.3 mm thick PDMS membranes, the survival rate became lower (around 60%). In future work, we will consider not only oxygen permeability but also other factors necessary for the development of chick embryos for longer incubation periods. For example, it has been reported by Ono et al. that until about Day 10 of incubation, the yolk was the only source of calcium for the development of chick embryos [54], and Kamihira et al. reported that calcium powder from normal eggshell was a crucial factor for achieving a longer survival period and increased hatching rate ability of chick embryos [20]. Therefore, we will try to add powder constituents to cubic eggshells in order to increase the viability of chick embryos. In addition, by fabricating the small patterns on the surface of the cubic eggshell using microfabrication techniques [55–57], the release ratio of the mixed constituents can be designed and controlled.

Blood vessel formation on the lateral-side membrane can be spatially induced by attaching polystyrene plates onto it to create oxygen non-permeable areas, which demonstrated an advantage of our design method. However, we did not quantitatively investigate the parameters such as the blood vessel diameter or the branching degree of the blood vessel network formed on the lateral-side membrane. Therefore, the details of the blood vessels on the patterned lateral-side membranes, which may affect the oxygen transportation to the heart of chick embryos, are still unknown. As a follow-up study, we will apply microfabrication techniques to create surface patterning of PDMS on the lateral-side membrane and locally change the membrane thickness (oxygen permeation). These techniques will enable us to precisely control parameters such as blood vessel branching and blood flow direction.

In this study, we proposed a generalized design method of artificial eggshell which focused on the oxygen permeability, and further, we showed a fabrication example by using two different types of materials to realize the artificial eggshell. The design approach based on Equation (1) shown under the Design of the artificial (cubic) eggshell in the article could be used for different sizes of avian embryos, such as Japanese quail [20]. We believe that “Egg-in-Cube” with a functionally-modified surface will further demonstrate the advantages of our approach.

## Conclusions

Here, a specific design and a fabrication method for artificial eggshells using an oxygen-permeable membrane surface have been proposed. The ethical threshold for the use of and the cost of chick embryos are relatively lower than that of the application of other experimental animals commonly employed in biomedical and biological research. The 7-day viability of the chick embryos in the cube-shaped artificial eggshells prepared by combining a 4-mm-thick frame and a 0.1- or 0.3-mm thick membrane was confirmed through basic experiments and observations. Blood vessel formation was observed not only on the top face but also on the lateral membranes of the cubic eggshells, which formed a complicated spatiotemporal blood vessel network. In addition, the pattern formation of blood vessels was designed by controlling the oxygen permeation volume. In other words, using the eggshell with specifically-designed surface, the objective of chick embryo culturing, observation, and operation in a lab environment was achieved, because the membrane surface is transparent and has controllable oxygen permeability and durability. These results suggest that the proposed approach of cubic eggshell design has huge potential as a functional platform for wide range of research fields such as drug screening, medical training, and educational programs.

## Supporting Information

S1 Fig Fabrication of a cubic eggshell and transfer of egg contents. (1) A hollow cubic frame was manufactured using a polymer material PC (PCP1609A, Takiron Co., Ltd.) by machining (α-T14iF, FANUC Co., Ltd.). (2) PDMS membranes with thickness were fabricated on the glass substrate by using a mixture of PDMS and a curing agent (Sylgard 184, Dow Corning) at a 10:1 ratio, and then cured on a hotplate (80°C, 20 min). (3) PDMS membranes were then attached to the hollow frame using PDMS glue and cured on the hotplate (80°C, 20 min), with the top surface unwrapped. (4) The cubic eggshell was filled with distilled water to confirm the lack of leakage. The eggshell was then sterilized by UV light (10 min) before transferring the egg contents. (5) The egg contents were transferred into the cubic eggshell. To avoid damage for the vascular network of chick embryo, 3-day-cultured eggs in the incubator were cracked and inserted to the cubic eggshell. (6) A piece of PDMS membrane was glued to the unwrapped face of the shell by using liquid PDMS. Finally, the chick embryos were incubated at 39°C at a relative humidity of 80%.
(TIF)

S2 Fig Thickness of membranes (*h*) used in the current study. The average thickness of membranes used in the current study were 0.12 (n = 42), 0.30 (n = 41), 0.48 (n = 30), 0.72 (n = 31), 1.02 (n = 26), and 1.31 (n = 30) mm for the desired values of membranes of 0.10, 0.30, 0.50, 0.70, 1.00, and 1.30 mm, respectively.
(TIF)

S3 Fig Analysis of heart rates in chick embryos. Movies of heart beats were obtained every 24 hours from day 4 to 7 (image size: 1920 × 1440, frame rate: 15 frames per second). (a) The filling and contraction of the cardiac chamber was analyzed frame by frame according to the blood flow movement. Images of a chick embryo (day 4) in a cubic eggshell fabricated using 0.3-mm-thick membranes are shown here (bar = 5 mm). (b) Then a waveform of average gray values within a rectangular area including the cardiac chamber was obtained from the movie. After that, heart rate was calculated from the waveform.
(TIF)

S4 Fig Image analysis of blood vessels on a patterned lateral-side membrane. (a) The lateral-side area occupied by albumen and the bottom edge of blood vessel network was traced manually, and then the parameters (area and height) of blood vessel network and the area occupied by albumen were obtained using the binarized image. (b) Definition of blood vessel area ratio (*A*_*b*_) and height ratio (*H*_*b*_).
(TIF)

S1 Movie Multi-directional observation of chick embryo. (MP4)

S2 Movie High-accessibility to chick embryo. (MP4)

S3 Movie Durability test for cubic eggshells. We tested the durability of the cubic eggshells fabricated using 0.1- and 0.3-mm-thick PDMS membranes. The movie shows that the cubic eggshell fabricated using a 0.1-mm-thick membrane was not durable for the insertion of surgical tools. On the other hand, chick embryos in the eggshell fabricated using a 0.3-mm-thick PDMS membrane could be manipulated from multiple directions, without creating a cleft on the membrane. This result demonstrates that the cubic eggshell fabricated using a 0.3-mm-thick membrane is appropriate to realize high accessibility.
(MP4)

S4 Movie Blood flowing observation using cubic eggshell. Blood flow in the blood vessels adhering on the lateral side of the cubic eggshell is shown here. The movie was obtained on day 7.
(MP4)
