# Supplementary figures and images for: Egg-in-Cube: Design and Fabrication of a Novel Artificial Eggshell with Functionalized Surface
Source: PLoS One. 2015 Mar 13;10(3):e0118624. doi: 10.1371/journal.pone.0118624 (PMC4359160; doi:10.1371/journal.pone.0118624)

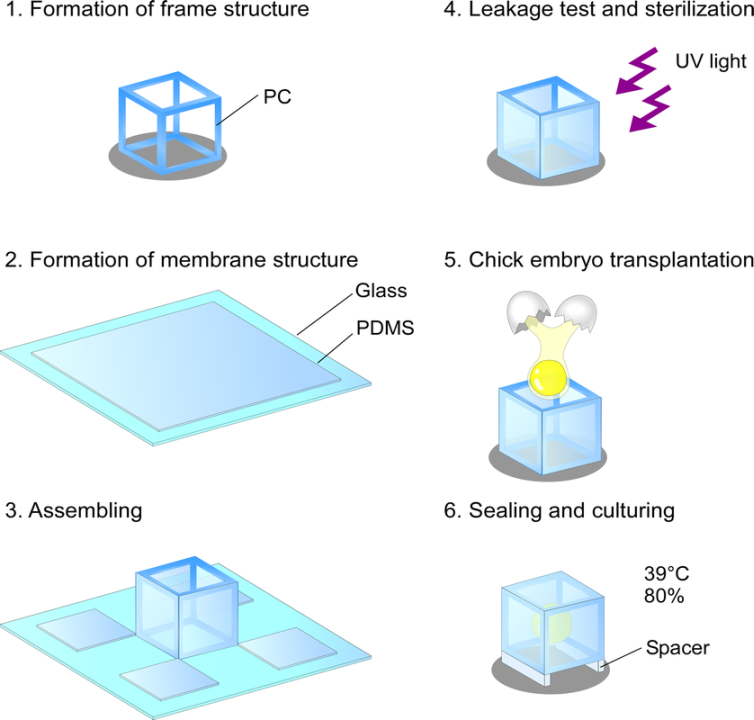

Supplement: S1 Fig — (1) A hollow cubic frame was manufactured using a polymer material PC (PCP1609A, Takiron Co., Ltd.) by machining (α-T14iF, FANUC Co., Ltd.). (2) PDMS membranes with thickness were fabricated on the glass substrate by using a mixture of PDMS and a curing agent (Sylgard 184, Dow Corning) at a 10:1 ratio, and then cured on a hotplate (80°C, 20 min). (3) PDMS membranes were then attached to the hollow frame using PDMS glue and cured on the hotplate (80°C, 20 min), with the top surface unwrapped. (4) The cubic eggshell was filled with distilled water to confirm the lack of leakage. The eggshell was then sterilized by UV light (10 min) before transferring the egg contents. (5) The egg contents were transferred into the cubic eggshell. To avoid damage for the vascular network of chick embryo, 3-day-cultured eggs in the incubator were cracked and inserted to the cubic eggshell. (6) A piece of PDMS membrane was glued to the unwrapped face of the shell by using liquid PDMS. Finally, the chick embryos were incubated at 39°C at a relative humidity of 80%. (TIF) [file pone.0118624.s001.tif]

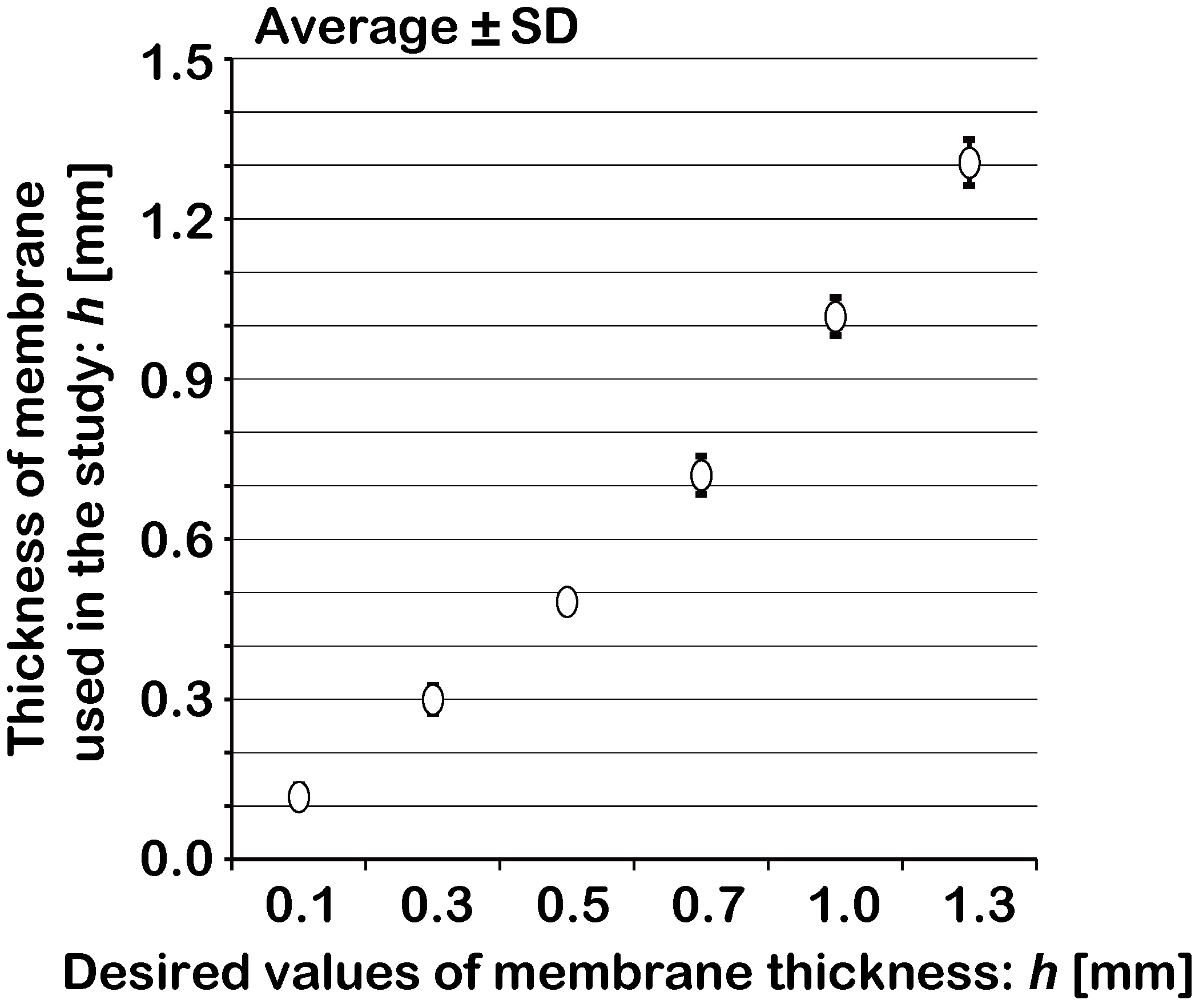

Supplement: S2 Fig — The average thickness of membranes used in the current study were 0.12 (n = 42), 0.30 (n = 41), 0.48 (n = 30), 0.72 (n = 31), 1.02 (n = 26), and 1.31 (n = 30) mm for the desired values of membranes of 0.10, 0.30, 0.50, 0.70, 1.00, and 1.30 mm, respectively. (TIF) [file pone.0118624.s002.tif]

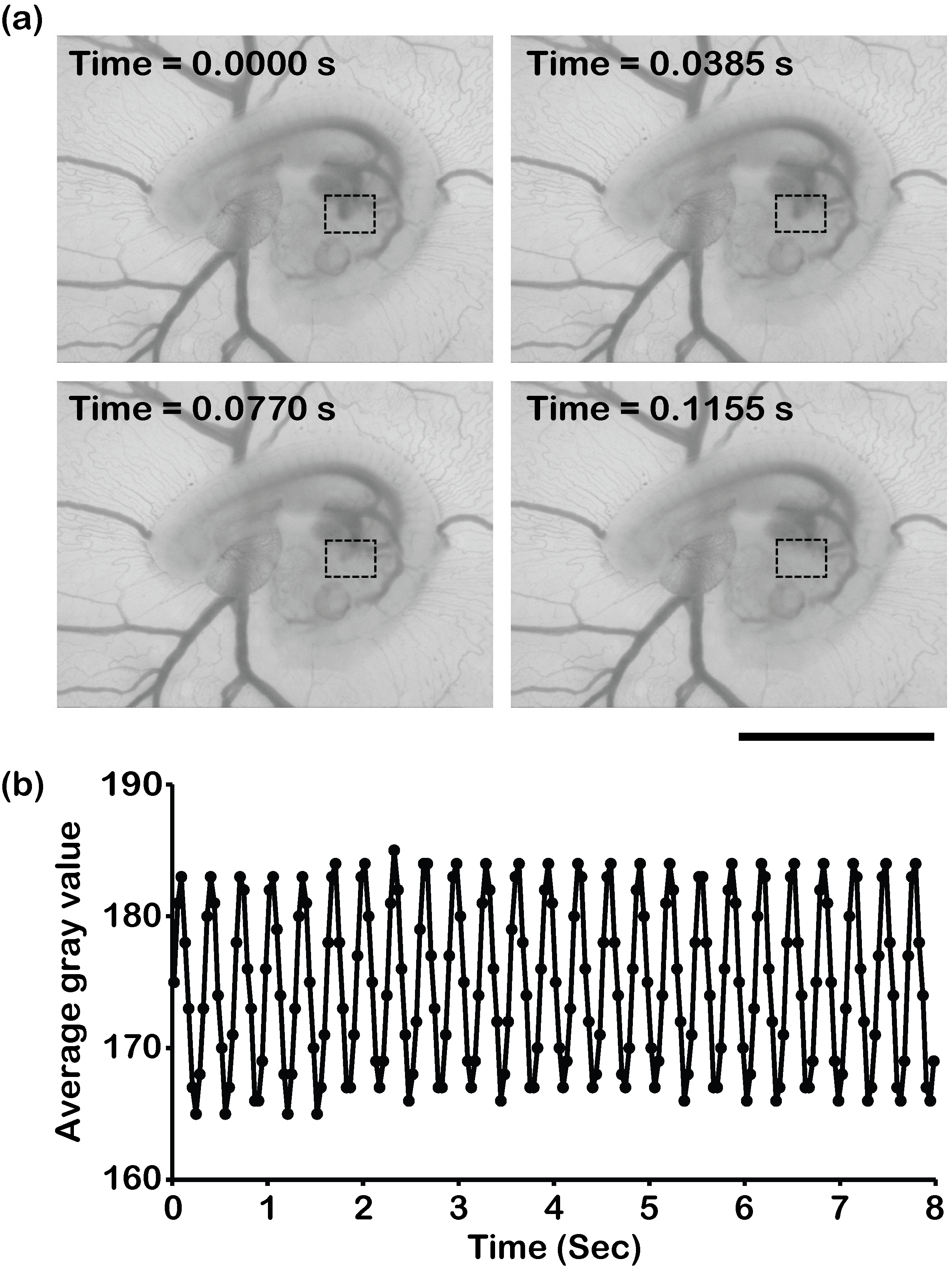

Supplement: S3 Fig — Movies of heart beats were obtained every 24 hours from day 4 to 7 (image size: 1920 × 1440, frame rate: 15 frames per second). (a) The filling and contraction of the cardiac chamber was analyzed frame by frame according to the blood flow movement. Images of a chick embryo (day 4) in a cubic eggshell fabricated using 0.3-mm-thick membranes are shown here (bar = 5 mm). (b) Then a waveform of average gray values within a rectangular area including the cardiac chamber was obtained from the movie. After that, heart rate was calculated from the waveform. (TIF) [file pone.0118624.s003.tif]

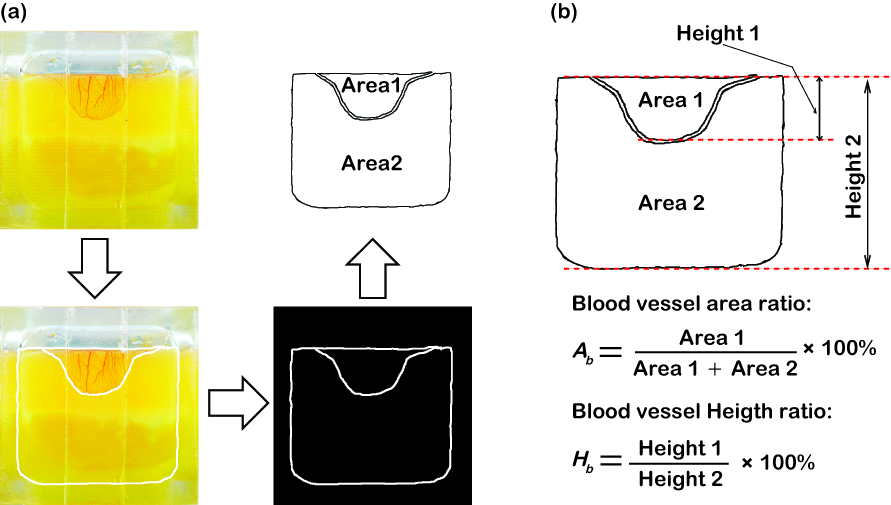

Supplement: S4 Fig — (a) The lateral-side area occupied by albumen and the bottom edge of blood vessel network was traced manually, and then the parameters (area and height) of blood vessel network and the area occupied by albumen were obtained using the binarized image. (b) Definition of blood vessel area ratio (A b) and height ratio (H b). (TIF) [file pone.0118624.s004.tif]
